# Effects of Extract from *Mangifera indica* Leaf on Monosodium Urate Crystal-Induced Gouty Arthritis in Rats

**DOI:** 10.1155/2012/967573

**Published:** 2012-11-19

**Authors:** Yan Jiang, Xiao-Ying You, Kong-Long Fu, Wan-Le Yin

**Affiliations:** People's Hospital of Zhengzhou, Henan Province, Zhengzhou 450003, China

## Abstract

The leaves of *Mangifera indica* L. (Anacardiaceae) is used as a medicinal material in traditional herb medicine for a long time in India, China, and other Eastern Asian countries. Our present study investigated the therapeutic effects of the ethanol extract from *Mangifera indica* (EMI) in rat with monosodium urate (MSU) crystals-induced gouty arthritis. Effects of EMI (50, 100, and 200 mg/kg, p.o.) administrated for 9 days on the ankle swelling, synovial tumor necrosis factor-alpha (TNF-**α**), and interleukin-1beta (IL-1**β**) levels were assessed in MSU crystal rat. Data from our study showed that rat with gouty arthritis induced by MSU crystal demonstrated an elevation in ankle swelling, synovial TNF-**α**, IL-1**β** mRNA, and protein levels. Oral administration of 100 and 200 mg/kg EMI for 9 days reversed the abnormalities in ankle swelling, synovial TNF-**α**, IL-1**β** mRNA, and protein levels. The results indicated that the beneficial antigouty arthritis effect of EMI may be mediated, at least in part, by inhibiting TNF-**α** and IL-1**β** expression in the synovial tissues. Our study suggests that *Mangifera indica* and its extract may have a considerable potential for development as an anti-gouty arthritis agent for clinical application.

## 1. Introduction

Gouty arthritis, a chronic disease characterized by hyperuricemia, affects the joints, particularly of the big toe, due to the body losing its ability to metabolize uric acid [1]. Monosodium urate (MSU) crystals are among the most potent proinflammatory stimuli, and an innate immune inflammatory response to the crystal surface is intimately involved in the pathology of gouty arthritis [2]. Cell activation by MSU microcrystals is a central feature of acute gouty arthritis and proinflammatory microcrystals can interact with all of the major synovial cell types, including neutrophils, monocytes/macrophages, and fibroblast-like (type B) synoviocytes [3, 4]. In monocytes, for example, microcrystals stimulate the synthesis of a number of proinflammatory cytokines, such as interleukin-1 (IL-1), IL-6, IL-8, and tumor necrosis factor-alpha (TNF-*α*) [4]. Particularly, it is well accepted that gouty arthritis is primarily driven by TNF-*α* and IL-1*β* [5, 6].

Besides synthetic chemical medicine, herbal therapy is another effective alternative to treat gout and related gouty arthritis. *Mangifera indica* L. (Anacardiaceae), the largest fruit-tree found in the wild in India and cultivated varieties, has been introduced to other warm regions of the world. Today, the medicinal purposes of *Mangifera indica* leaf have been widely studied. For example, it has recently been reported that extract of *Mangifera indica* leaf inhibited lipid peroxidation [7], exerted antifungal activity [8], and exhibited antiulcerogenic action [9].

However, antigouty arthritis effects of *Mangifera indica* leaf have not been reported so far. This study was, therefore, undertaken to examine the anti-inflammatory effect of *Mangifera indica* leaf against MSU crystal-induced inflammation, an experimental model for gouty arthritis in rat. Furthermore, we focused on the effect of *Mangifera indica* leaf on the induction of inflammatory cytokines TNF-*α* and IL-1*β* in gouty arthritis to investigate its anti-inflammatory mechanism.

## 2. Materials and Methods

### 2.1. Plant Material and Preparation of Extract

The leaves of *Mangifera indica* were collected from Nanning (Guangxi Province, China) in the month of April 2011, and identified by Professor Sui-Qing Cheng, Henan University of Traditional Chinese Medicine. Voucher specimen (no. 11015) is kept in the herbarium of Hospital. Dried powdered leaves of *Mangifera indica* (500 g) were extracted with ethanol (2.5 L) and were allowed to stand at room temperature for about 16 h. The percolate was collected. This process of extraction was repeated three times. The solutions were combined, filtered, concentrated under reduced pressure, and lyophilized into powders (EMI), and the weight of extract obtained was 48.6 g.

### 2.2. Animals

Male Sprague-Dawley rats (240 ± 20 g) were purchased from the Laboratory Animal Center of Henan Province. Animals were singly housed (320 × 180 × 160 cm) under a normal 12 h light/dark schedule with the lights on at 07:00 a.m and had free access to tap water and food pellets. Ambient temperature and relative humidity were maintained at 22 ± 2°C and at 55 ± 5% and were given a standard chow and water *ad libitum* for the duration of the study. All experimental protocols described in this study were conducted in accordance with the Institutional Animal Care Committee at People's Hospital of Zhengzhou.

### 2.3. Drugs and Treatment Schedule

MSU was dissolved in boiling water containing 1N NaOH. After the pH was adjusted to 7.2, the solution was cooled gradually by stirring at room temperature. The crystals were collected by centrifugation at 3000 ×g for 2 min at 4°C. Then the crystals were evaporated and sterilized by heating at 180°C for 2 hours and stored in a sterile environment until use. Finally, the MSU crystals were suspended at 20 mg/mL in sterile, endotoxin-free PBS and verified to be free of detectable lipopolysaccharide (LPS) contamination (<0.0625 endotoxin U/mL) by the limulus amebocyte lysate assay according to the Pharmacopoeia of the People's Republic of China [10].

The MSU crystal-induced gouty arthritis used was the same as described in detail elsewhere [11], with some modification. Briefly, rats were divided into six groups—each comprising of seven animals. Group I served as a control group. In group II, gouty arthritis was induced by MSU crystal and treated by vehicle (Model group). Group III-V comprised of MSU crystal-induced rats which were treated with EMI (50, 100, and 200 mg/kg) and group VI monosodium crystal-induced rats were treated with indomethacin (5 mg/kg). EMI and indomethacin were oral administered once a day for 9 days. Arthritis was conducted on Day 7, 60 min after EMI administration. Arthritis was induced in anesthetized (10% Chloral Hydrate, 0.3 mL/100 g, ip) rats by injecting 0.1 mL MSU crystal (100 mg/mL) in the left ankle joint. The development of arthritis was assessed by measuring the size of the joint with MK-550 volume meter immediately before the injection. Subsequent measurements of the same ankle joint were carried out at 2, 4, 8, 12, 24, and 48 hours after the injection.

### 2.4. Real-Time PCR for Detecting mRNA of TNF-*α* and IL-1*β*

Total RNA was isolated from synovial tissue using trizol reagent following the manufacturer's instructions. Reverse transcription was performed using M-MLV reverse transcriptase for cDNA synthesis. The primer was as below: TNF-*α* (Forward: 5′-ACTTGTGCCTATCTGCTT-3′, Reverse: 5′-AGTTCATAAATCCCTCCT-3′, 470 bp), IL-1*β* (Forward: 5′-AGGCTCCGAGATGAACAA-3′, Reverse: 5′-AAGGCATTAGAAACAGTCC-3′, 464 bp). The amplificatory reactions were performed with 10 *μ*L of double concentrated SYBR Green realtime PCR master mix, each 0.4 *μ*L of 10 *μ*M primer and 1 *μ*L cDNA in a final volume of 25 *μ*L. The cycle threshold (Ct) values for the reactions of relative quantification PCR was defined as the cycle of PCR which amplified the product that was first detected. The expression of TNF-*α* and IL-1*β* mRNA was analyzed by the 2^−ΔΔCt^. The results were normalized to the mRNA expression level of GAPDH in each sample.

### 2.5. Determination of TNF-*α* and IL-1*β* Levels

Synovial tissue was cut into tubes and homogenized in lysis buffer (Boster, Wuhan, China). The homogenate was centrifugated at 16000 ×g for 30 min at 4°C, and the supernatant was collected and stored at −80°C until assay. Protein levels of samples were measured using the Lowry Method [12].

TNF-*α* and IL-1*β* levels were measured using ELISA kit according to the manufacturer's instructions (Boster, Wuhan, China).

### 2.6. Statistical Analyses

All data were expressed as mean ± S.E.M. To compare experimental and control groups, we used one-way ANOVA, followed by post hoc Dunnett's test. Data of ankle swelling were analyzed using a repeated ANOVA with treatment as between factor and time (hours) as within factor. A value of *P* < 0.05 was considered statistically significant for analysis.

## 3. Results

### 3.1. Effects of EMI on Ankle Swelling in MSU Crystal-Gouty Arthritis Rats

**Figure 1:**
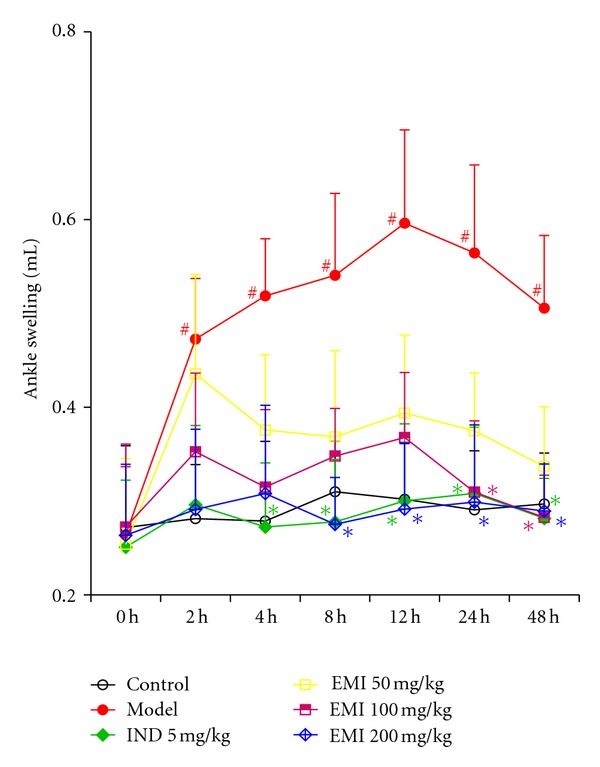
Effects of EMI on ankle swelling in MSU crystal-induced gouty arthritis rats. Rats were pretreated with either vehicle, EMI (50, 100, and 200 mg/kg, p.o.), or positive drug indomethacin (5 mg/kg). Ankle swellings were evaluated at different time points. Data were expressed as the mean ± S.E.M. (*n* = 7). ^#^ *P* < 0.05 versus vehicle-control group (Control); *P < 0.05 versus vehicle with MSU crystal (Model).

As shown in Figure 1, a one-way ANOVA revealed that baseline ankle swelling did not differ between the control and MSU crystal-induced groups (0 h). From 0 h to 48 h, a repeated ANOVA with MSU crystal as independent factor and hour as repeated factor, revealed no statistically significant effects of MSU crystal, hour, and MSU crystal × hour interaction on ankle swelling. Further separate repeated ANOVA, with treatment as independent factor and hour as repeated factor, revealed a gradual ankle swelling reduction in MSU crystal-induced rats [*F*(4, 30) = 8.33, *P* < 0.01]. Post hoc test showed that both EMI (50 mg/kg: *P* < 0.05; 100 mg/kg: *P* < 0.01; 200 mg/kg: *P* < 0.01, resp.) and indomethacin (*P* < 0.01) decreased the ankle swelling levels compared to the model group.

In addition, a separate one-way ANOVA showed that MSU crystal increased the ankle swelling levels compared to control group in 2 h [*F*(1, 12) = 4.88, *P* < 0.05], 4 h [*F*(1, 12) = 5.25, *P* < 0.05], 8 h [*F*(1, 12) = 5.06, *P* < 0.05], 12 h [*F*(1, 12) = 6.45, *P* < 0.05], 24 h [*F*(1, 12) = 5.87, *P* < 0.05], and 48 h [*F*(1, 12) = 4.85, *P* < 0.05]. Post hoc test revealed that 5 mg/kg indomethacin (*P* < 0.05) reduced the ankle swelling after 4 h. After 8 h and 12 h, 200 mg/kg EMI (*P* < 0.05, *P* < 0.05, resp.) and 5 mg/kg indomethacin (*P* < 0.05, *P* < 0.05, resp.) markedly decreased the ankle swelling, while 100 (*P* < 0.05, *P* < 0.05, resp.), 200 (*P* < 0.05, *P* < 0.05, resp.) mg/kg EMI, and indomethacin (*P* < 0.05, *P* < 0.05, resp.) decreased the ankle swelling after 24 h and 48 h.

### 3.2. Effects of EMI on TNF-*α* and IL-1*β* mRNA Expression in Synovial Tissues

**Figure 2:**
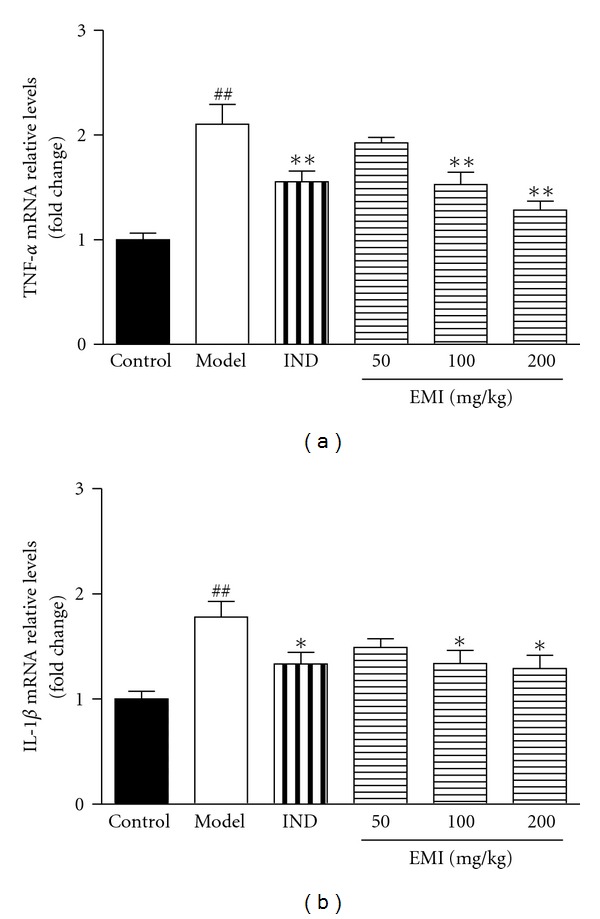
Effects of EMI on synovial tissue TNF-*α* and IL-1*β* mRNA expression in MSU crystal-induced gouty arthritis rats. Data were expressed as the mean ± S.E.M. (*n* = 7). ^##^ *P* < 0.01 versus vehicle-control group (Control); *P < 0.05 and **P < 0.01 versus vehicle with MSU crystal (Model).

As shown in Figure 2, MSU crystal induced a remarkable elevation of TNF-*α* and IL-1*β* mRNA expressions in synovial tissues (*P* < 0.01, *P* < 0.01, resp.). Post-hoc analysis indicated that EMI (100 and 200 mg/kg) and indomethacin (5 mg/kg) decreased TNF-*α* (*P* < 0.01, *P* < 0.01, *P* < 0.01, resp.) and IL-1*β* (*P* < 0.05, *P* < 0.05, *P* < 0.05, resp.) mRNA expressions.

### 3.3. Effects of EMI on TNF-*α* and IL-1*β* Levels in Synovial Tissues

**Figure 3:**
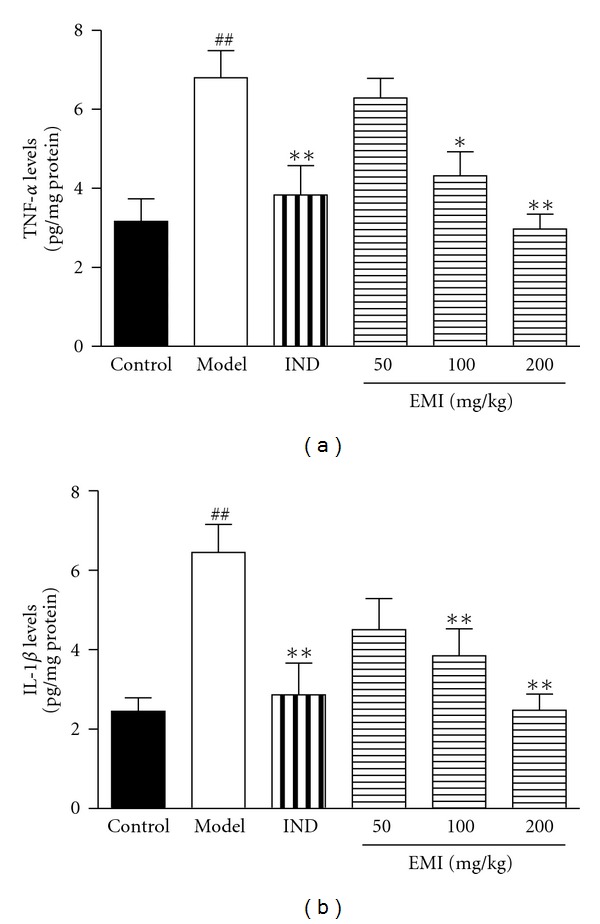
Effects of EMI on synovial tissue TNF-*α* and IL-1*β* levels in MSU crystal-induced gouty arthritis rats. Data were expressed as the mean ± S.E.M. (*n* = 7). ^##^ *P* < 0.01 versus vehicle-control group (Control); *P < 0.05 and **P < 0.01 versus vehicle with MSU crystal (Model).

As shown in Figure 3, MSU crystal induced a significant increase of TNF-*α* and IL-1*β* levels in synovial tissues (*P* < 0.01, *P* < 0.01, resp.). Post-hoc analysis indicated that EMI decreased TNF-*α* (100 mg/kg: *P* < 0.05; 200 mg/kg: *P* < 0.01, resp.) and IL-1*β* (100 mg/kg: *P* < 0.01; 200 mg/kg: *P* < 0.01, resp.) levels. In addition, the positive drug indomethacin (5 mg/kg) also reduced the cytokines levels (*P* < 0.01, *P* < 0.01, resp.) compared to the model group.

## 4. Discussion

Over the years, increasing proportion of patients with gouty arthritis and other metabolic disorders are resorting to complementary and alternative medicine for their health needs [13]. Natural plant products comprise one of the most popular complementary and alternative medicines for inflammatory disorders [14, 15]. These herbal medicines belong to diverse traditional systems of medicine, including traditional Chinese medicine, Kampo, and Ayurvedic medicine. More important, pharma industry has seen a shift from the search for specifically target drugs to the pursuit of combination or herb therapies that comprise more than one active ingredient [16]. In our present study, we assessed the antigouty arthritis effect of the herb extract, EMI in rats with MSU crystal-induced gouty arthritis.

Gout is a medical condition usually characterized by recurrent attacks of acute inflammatory arthritis—a red, tender, hot, and swollen joint. A definitive diagnosis of gout arthritis is based upon the identification of MSU crystals in synovial fluid or a tophus [17]. In our study, EMI treatment (200 mg/kg) reversed MSU crystal-induced elevation in ankle swelling, and the onset of amelioration, that is, a significant decrease in ankle swelling was seen at hour 8 and remained in subsequent hours. In addition, 100 mg/kg EMI only reduced the ankle swelling after 24 h and 48 h. However, EMI at 50 mg/kg could not affect the high levels of ankle swelling induced by MSU crystal. In line with our study, a previous study performing the same protocol showed that MSU crystal induced the abnormality of ankle swelling, and indomethacin could reverse the changes [18]. As a result, the findings present from our study indicated that EMI, in addition to having many previously described biological activities (seen in Introduction Section), may be a potent antigouty arthritis agent.

It is well accepted that proinflammatory cytokines help to propagate a local or systemic inflammatory process [19]. Cytokines such as TNF-*α* and IL-1*β* have been found in symptomatic joints of arthritic patients and has been implicated in acute and chronic arthritic diseases [20]. Upon contact with host cells, MSU induces a set of membrane events that trigger related signaling pathway, phagocytosis, and cytokine production. Having entered the cell, MSU further triggers inflammasome activation and induces the production of TNF-*α* and IL-1*β*, likely inducing a full spectrum of inflammation [21]. TNF-*α* and IL-1*β* by monocytes/macrophages upon phagocytosis of MSU crystal play a central role in the initiation and propagation of the inflammatory response [22, 23]. Therefore, the treatment of inflammation is the major therapeutic approache against gouty arthritis [24]. Our results demonstrated that EMI at 100 and 200 mg/kg not only reduced the synovial TNF-*α* and IL-1*β* levels induced by MSU crystal, but also decreased their mRNA expression. Indomethacin, a drug often used as first-line therapies for acute inflammation in gouty arthritis, also reduced the TNF-*α* and IL-1*β* mRNA and protein levels in rats induced by MSU crystal. Therefore, similar to the positive drug indomethacin, EMI may be served as an antigouty arthritis agent mediated by inhibition of cytokines expression such as TNF-*α* and IL-1*β*.

In addition, although the reports claim that MSU crystals are the inducers of proinflammatory cytokines production [21–23], it should be noted that the cytokines induction can be also evoked by endotoxin LPS [25–27]. Moreover, the concentrations of cytokines induced by MSU were much lower than those achieved by stimulation with LPS [28, 29]. More importantly, the studies found that the production of the cytokines induced by MSU crystal can be synergistically enhanced by LPS [28, 29]. In this respect, the low stimulation of cytokines by MSU alone and the strong synergy with LPS has additional implications. Thus, studies reporting strong induction of cytokines by MSU alone should be interpreted with caution if the LPS contamination was not excluded, as even minor LPS contamination could lead to erroneous results [28]. Therefore, the limulus amebocyte lysate assay was used in our study to rule out the interference by LPS. The results (<0.0625 endotoxin U/mL) verified that MSU crystal-induced proinflammatory cytokines overexpression was not due to trace endotoxin contamination.

## 5. Conclusion

The present results clearly demonstrated that EMI was able to significantly decrease ankle swelling in MSU crystal-induced gouty arthritis rats. This beneficial antigouty arthritis effect may be mediated, at least in part, by inhibiting TNF-*α* and IL-1*β* mRNA expression and protein levels in the synovial tissues. Further works, including clinical application, will be necessary to determine whether EMI produces a similar therapeutic efficacy as was observed in the present study.
